# MODOMICS: a database of RNA modification pathways. 2021 update

**DOI:** 10.1093/nar/gkab1083

**Published:** 2021-12-06

**Authors:** Pietro Boccaletto, Filip Stefaniak, Angana Ray, Andrea Cappannini, Sunandan Mukherjee, Elżbieta Purta, Małgorzata Kurkowska, Niloofar Shirvanizadeh, Eliana Destefanis, Paula Groza, Gülben Avşar, Antonia Romitelli, Pınar Pir, Erik Dassi, Silvestro G Conticello, Francesca Aguilo, Janusz M Bujnicki

**Affiliations:** Laboratory of Bioinformatics and Protein Engineering, International Institute of Molecular and Cell Biology in Warsaw, ul. Ks. Trojdena 4, PL-02-109 Warsaw, Poland; Laboratory of Bioinformatics and Protein Engineering, International Institute of Molecular and Cell Biology in Warsaw, ul. Ks. Trojdena 4, PL-02-109 Warsaw, Poland; Laboratory of Bioinformatics and Protein Engineering, International Institute of Molecular and Cell Biology in Warsaw, ul. Ks. Trojdena 4, PL-02-109 Warsaw, Poland; Laboratory of Bioinformatics and Protein Engineering, International Institute of Molecular and Cell Biology in Warsaw, ul. Ks. Trojdena 4, PL-02-109 Warsaw, Poland; Laboratory of Bioinformatics and Protein Engineering, International Institute of Molecular and Cell Biology in Warsaw, ul. Ks. Trojdena 4, PL-02-109 Warsaw, Poland; Laboratory of Bioinformatics and Protein Engineering, International Institute of Molecular and Cell Biology in Warsaw, ul. Ks. Trojdena 4, PL-02-109 Warsaw, Poland; Laboratory of Bioinformatics and Protein Engineering, International Institute of Molecular and Cell Biology in Warsaw, ul. Ks. Trojdena 4, PL-02-109 Warsaw, Poland; Laboratory of Bioinformatics and Protein Engineering, International Institute of Molecular and Cell Biology in Warsaw, ul. Ks. Trojdena 4, PL-02-109 Warsaw, Poland; Department of Cellular, Computational and Integrative Biology, University of Trento, Via Sommarive 9, 38123 Trento, Italy; Department of Molecular Biology, Umeå University, SE-901 85 Umeå, Sweden; Wallenberg Centre for Molecular Medicine, Umeå University, SE-901 85 Umeå, Sweden; Department of Bioengineering, Gebze Technical University, 41400 Kocaeli, Turkey; Core Research Laboratory, ISPRO—Institute for Cancer Research, Prevention and Clinical Network, 50139 Firenze, Italy; Department of Medical Biotechnologies, Università di Siena; Department of Bioengineering, Gebze Technical University, 41400 Kocaeli, Turkey; Department of Cellular, Computational and Integrative Biology, University of Trento, Via Sommarive 9, 38123 Trento, Italy; Core Research Laboratory, ISPRO—Institute for Cancer Research, Prevention and Clinical Network, 50139 Firenze, Italy; Institute of Clinical Physiology, National Research Council, 56124 Pisa, Italy; Department of Molecular Biology, Umeå University, SE-901 85 Umeå, Sweden; Wallenberg Centre for Molecular Medicine, Umeå University, SE-901 85 Umeå, Sweden; Laboratory of Bioinformatics and Protein Engineering, International Institute of Molecular and Cell Biology in Warsaw, ul. Ks. Trojdena 4, PL-02-109 Warsaw, Poland

## Abstract

The MODOMICS database has been, since 2006, a manually curated and centralized resource, storing and distributing comprehensive information about modified ribonucleosides. Originally, it only contained data on the chemical structures of modified ribonucleosides, their biosynthetic pathways, the location of modified residues in RNA sequences, and RNA-modifying enzymes. Over the years, prompted by the accumulation of new knowledge and new types of data, it has been updated with new information and functionalities. In this new release, we have created a catalog of RNA modifications linked to human diseases, e.g., due to mutations in genes encoding modification enzymes. MODOMICS has been linked extensively to RCSB Protein Data Bank, and sequences of experimentally determined RNA structures with modified residues have been added. This expansion was accompanied by including nucleotide 5′-monophosphate residues. We redesigned the web interface and upgraded the database backend. In addition, a search engine for chemically similar modified residues has been included that can be queried by SMILES codes or by drawing chemical molecules. Finally, previously available datasets of modified residues, biosynthetic pathways, and RNA-modifying enzymes have been updated. Overall, we provide users with a new, enhanced, and restyled tool for research on RNA modification. MODOMICS is available at https://iimcb.genesilico.pl/modomics/.

## INTRODUCTION

RNA building blocks include not only the four basic A, U, C and G ribonucleosides/ribonucleotides, but >150 chemically altered residues. RNA modifications have been identified in all types of RNA molecules, including transfer RNA (tRNA), ribosomal (rRNA), and messenger (mRNA), as well as various short and long non-coding RNAs (lncRNAs). Many of these modified residues play important roles in RNA metabolism, including RNA structure formation, stability and dynamics, RNA splicing, polyadenylation, transport, localization, and translatability [recent reviews: (1–3)]. They can also be important for RNA interactions with other molecules, particularly with proteins and ribonucleoproteins (4).

Most of the naturally occurring RNA modifications are introduced post-transcriptionally by various enzymes, and modification reactions form very complex pathways, leading to hypermodified residues (5). Not all RNA modifications can be synthesized by the organisms in which they are found but can be delivered as a nutrient, e.g., by the microbiome (6,7). Furthermore, some RNA modifications are reversible, i.e., the modified residues can be enzymatically unmodified, allowing a quick post-transcriptional response to changing cellular or environmental conditions (2,8–10). These epitranscriptomic modifications can affect the RNA itself (e.g. directly affecting the RNA structure) or need specialized readers to fulfill their purpose.

The progress in high-throughput sequencing and the advances in methods for the discovery of modified residues at the transcriptome scale have led to the development of the new field of research termed ‘epitranscriptomics’ (11–13). Moreover, the growing amount of information concerning modified nucleosides in RNA has improved the understanding of their function. Changes in RNA modifications have been recognized as the cause of various human diseases, including cancer, immune disorders, and neuromuscular defects (14–17). Consequently, improved understanding of the properties of RNAs with modified residues has allowed their use to develop RNA therapeutics, e.g., to improve the properties of RNA vaccines (18–20).

## DATABASE CONTENT

**Figure 1.**
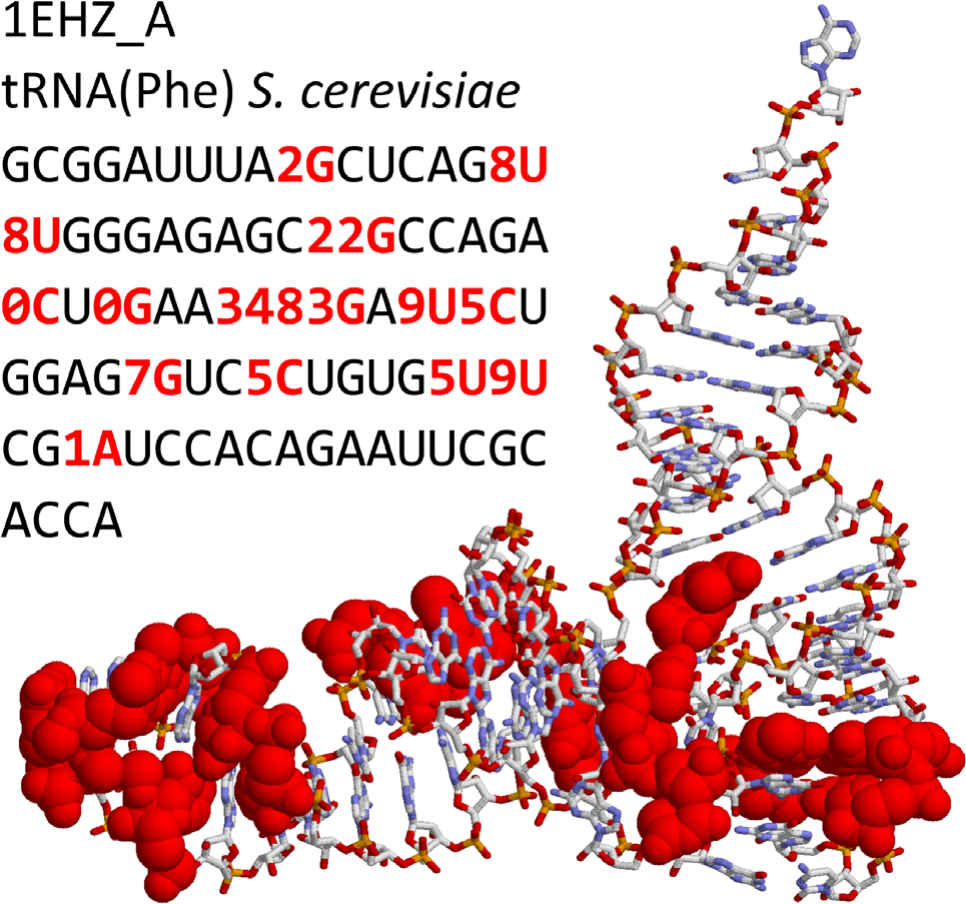
Example of an experimentally determined 3D structure of RNA with modified residues. Sequences of experimentally determined RNAs containing modifications are now imported to MODOMICS from the RSCB PDB database, with links to the original structural data. Here, the sequence of tRNA(Phe) from *S. cerevisiae* (RCSB ID 1ehz chain A) is shown, with modified ribonucleotide residues colored in red. In the corresponding sequence modified residues are indicated with the MODOMICS ribonucleoside codes and are also colored in red.

**Figure 2.**
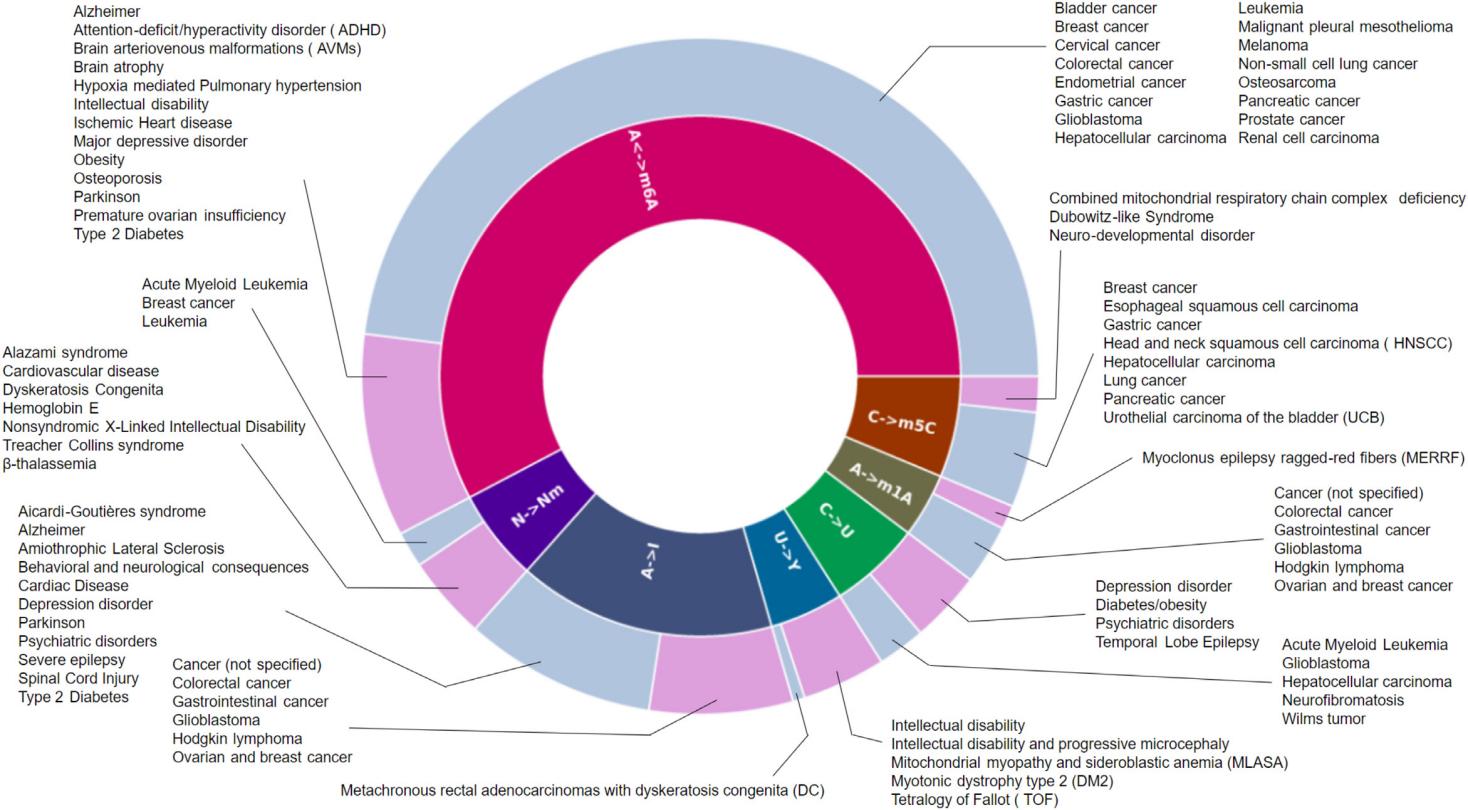
Links between the disturbance of RNA modification and disease. Cancer-related diseases are indicated in grey, other diseases are indicated in magenta.

As in the previous release (21), MODOMICS hosts a catalog of modified residues, enzymes and guide RNAs responsible for individual reactions, RNA modification pathways, sequences of modified RNAs, a catalog of ‘building blocks’ for chemical synthesis of modified RNA, and other associated data such as relevant publications. The new version of MODOMICS includes updates on datasets of modified residues, biosynthetic pathways, and RNA-modifying enzymes with an integrated search engine for similarly chemically modified residues. New features for nucleotide 5′-monophosphate residues to match the nucleoside counterparts and sequences of experimentally determined RNA structures with modified residues have been added (an example illustrated in Figure 1), enabling MODOMICS to be linked to the RCSB Protein Data Bank database (rcsb.org) (22). Additionally, we have included a new section Human Diseases, which encompasses links of RNA modifications to different diseases, including cancer and neurodegeneration (Figure 2).

Last but not least, both the backend and the frontend of MODOMICS were re-developed. The current version of MODOMICS is based on the Django Python framework v 3.1. The user interface was completely reconstructed, and the Pathways page was re-implemented in javascript, replacing the obsolete Adobe Flash, thus allowing for rendering the visualization in most browsers. The pathways can now also be represented using a network graph layout in addition to the classical hierarchical graph representation. A completely new application programming interface (API) system (including other computational resources) allows the users to interact programmatically with the modification data present in the database. This API returns a response based on the user request formatted as plain text, comma-separated values, or JSON format.

### New section: human diseases

MODOMICS was expanded to include a new section that provides associations between RNA modifications and human diseases. The main entry point provides a list of observations linking the disturbance in a process related to RNA modification to a pathological condition in humans. Many of such entries include associations of a mutation in human genes encoding RNA modification enzymes with a disease or, more generally, a pathophysiological condition. Cases of dysregulated expression of RNA modification genes were also included. The knowledge of the impact of RNA modifications on human health and disease expands rapidly, and new studies linking RNA modification status to disease conditions, e.g., cancer, appear almost every day. In addition, entries of the human RNA-modifying enzymes list disease conditions associated with a given enzyme malfunction or misregulation. Considering the fast growth of the epitranscriptomic field, this section of MODOMICS does not intend to provide a comprehensive and up-to-date view of all studies that link epitranscriptomics to health conditions. Instead, we aim to highlight the association between RNA modification pathways and disease. MODOMICS indicates the modifications linked to specific disease conditions, rather than broadly focusing on disease and comprehensively listing all kinds of modification disturbance observed. We invite the scientific community to contact us to expand the catalog of epitranscriptomics-disease links in MODOMICS.

### Updated modifications section

172 new modified residues were added to the database. Previously, the catalog of modifications in MODOMICS included mostly ribonucleosides and only a few ribonucleotides associated with modifications of the 5′ and 3′ termini, e.g., various caps. For the current release, this catalog was expanded to include nucleotide 5′-monophosphate counterparts of all ribonucleosides. This expansion enabled MODOMICS to be linked to the ligand (LIG) entries in the RCSB Protein Data Bank database and establish a relationship between modified residues in MODOMICS and the experimentally determined RNA structures in the RCSB database that contain modified residues. It must be emphasized that for one nucleoside residue, multiple nucleotide residues can exist that differ by the number and position of phosphates at 5′, 3′ and 2′ positions, e.g., G versus GMP, GDP, GTP, 2′3′-cyclic phosphate, etc. Only some of the possible nucleotide residues are relevant for consideration in MODOMICS. Currently, MODOMICS contains 180 nucleotide residues, 152 nucleoside residues and three bases (modified bases are exclusively in the queuosine pathway).

Chemical structures deposited in the MODOMICS database were manually curated. This process included the standardization of structures and correcting/adding stereochemical information. Chemistry-related information on the modified residues was substantially updated. Data on physicochemical properties, such as predicted octanol/water partition coefficient (log*P*), topological polar surface area (TPSA), and a number of hydrogen bond donors and acceptors (HBD and HBA) were added. Each chemical structure is now available for download in multiple formats (mol, mol2, sdf, SMILES, InChI, and pdb) as a two-dimensional flat structure (2D) or three-dimensional low-energy conformer (3D), generated using Open Babel (23). Each structure has a set of alternative tautomers precalculated and available for download or display (24). Also, predictions of sites in a molecule that are most liable to metabolism by cytochrome P450 are displayed for each modification molecule (25).

The page for an individual residue includes new and updated links to the external resources. The most important new additions are the RCSB Protein Data Bank database (links to structures, where the given moiety is a free ligand), Human Metabolome Database (26), PubChem (the world's largest collection of freely accessible chemical information) (27), and ChEMBL (a manually curated database of bioactive molecules with drug-like properties) (28). The chemical structures of the modified residues are now depicted in a unified way using the RDKit framework (http://www.rdkit.org).

### The chemical similarity of modified residues

MODOMICS now offers a structural comparison of modified residues. Search options include the exact search (to find structures exactly matching the provided structure), the substructure search (to find structures containing the provided fragment), and similarity search (to find structures similar to the provided one). The search structure can be provided by pasting the SMILES code (available from MODOMICS for all modified residues) or by drawing the structure in a molecular editor (29). The search is performed using OpenBabel FastSearch engine with the FP2 path-based fingerprint.

### Updated collection of proteins, enzymatic activities, and pathways

The MODOMICS collection of functionally characterized proteins involved in RNA modification is under constant development. 40 new proteins were added to the previous collection. Among new proteins that were added in this release are SARS Cov-2 NSP proteins involved in the capping of viral RNA (30–32) and a collection of decapping enzymes from Nudix, DXO, HIT and APAH-like families (33). Besides these newly characterized enzymes, data entries for many enzymes and associated pathways were updated.

### Future prospects

Thus far, MODOMICS has been mainly focused on individual modification sites and reactions studied by biochemical approaches. The advances of epitranscriptomics provide new datasets and databases that address the presence of modified residues across different transcriptomes. MODOMICS will continue to be focused on the biochemistry and biochemical pathways of RNA modifications and does not intend to become a primary resource for storing epitranscriptome-wide RNA modification data. On the other hand, MODOMICS will certainly need to store sequence information about experimentally validated sites of modified residues in a much larger group of transcripts. There are multiple projects and databases devoted to mapping various modifications on the transcriptome scale, and the evidence in the field is of variable quality. Hence, for the next release of MODOMICS, we plan to establish links to primary databases storing information about the sites of modifications. In this context, we plan to include the annotation of the individual modification sites, to distinguish information obtained from different primary sources, so the users could identify and select the data only from the sources they trust, e.g., obtained with two independent experimental approaches. Further, we plan to extend information related to the structural context of RNA modifications, to include non-natural modifications of RNA molecules present in experimentally determined 3D structures, update the visualization options, and refurbish the website to keep up with changing trends in web design. We also intend to renew data structures, make MODOMICS more compatible with other databases and web servers, facilitate automated data exchange, and introduce the ability to search sequences with strings that include modification codes.

## DATA AVAILABILITY

The data are accessible freely for research purposes at https://iimcb.genesilico.pl/modomics/.
